# The Ebola Outbreak: Catalyzing a “Shift” in Global Health Governance?

**DOI:** 10.1186/s12879-016-2016-y

**Published:** 2016-11-24

**Authors:** Tim K. Mackey

**Affiliations:** 1 Department of Anesthesiology, University of California, San Diego School of Medicine, San Diego, CA USA; 2 Department of Medicine, Division of Global Public Health, University of California, San Diego School of Medicine, San Diego, CA USA; 3 Global Health Policy Institute, 6256 Greenwich Drive, Mail Code: 0172X, San Diego, CA 92122 USA

**Keywords:** Ebola virus disease, Global health governance, WHO reform, International Health Regulations, Global health security agenda

## Abstract

**Background:**

As the 2014 Ebola virus disease outbreak (EVD) transitions to its post-endemic phase, its impact on the future of global public health, particularly the World Health Organization (WHO), is the subject of continued debate. Criticism of WHO’s performance grew louder in the outbreak’s wake, placing this international health UN-specialized agency in the difficult position of navigating a complex series of reform recommendations put forth by different stakeholders. Decisions on WHO governance reform and the broader role of the United Nations could very well shape the future landscape of 21st century global health and how the international community responds to health emergencies.

**Discussion:**

In order to better understand the implications of the EVD outbreak on global health and infectious disease governance, this debate article critically examines a series of reports issued by four high-level commissions/panels convened to specifically assess WHO’s performance post-Ebola. Collectively, these recommendations add increasing complexity to the urgent need for WHO reform, a process that the agency must carry out in order to maintain its legitimacy. Proposals that garnered strong support included the formation of an independent WHO Centre for Emergency Preparedness and Response, the urgent need to increase WHO infectious disease funding and capacity, and establishing better operational and policy coordination between WHO, UN agencies, and other global health partners. The recommendations also raise more fundamental questions about restructuring the global health architecture, and whether the UN should play a more active role in global health governance.

**Summary:**

Despite the need for a fully modernized WHO, reform proposals recently announced by WHO fail to achieve the “evolution” in global health governance needed in order to ensure that global society is adequately protected against the multifaceted and increasingly complex nature of modern public health emergencies. Instead, the lasting legacy of the EVD outbreak may be its foreshadowing of a governance “shift” in formal sharing of the complex responsibilities of global health, health security, outbreak response, and managing health emergencies to other international structures, most notably the United Nations. Only time will tell if the legacy of EVD will include a WHO that has the full support of the international community and is capable of leading human society in this brave new era of the globalization of infectious diseases.

## Background

In January 2016, the World Health Organization (WHO) declared that the devastating 2014 Ebola virus disease (EVD) outbreak in West Africa was finally coming to an end and in March officially announced it was no longer a public health emergency [1]. These developments came after nearly three years of unprecedented international cooperation to combat the largest Ebola outbreak in history, one that has claimed the lives of over 11,000 people and wrought social and economic devastation to Liberia, Sierra Leone, and Guinea, the three countries most heavily impacted. Re-emergence of EVD through detection of new case clusters after countries had been declared “Ebola Free” by WHO have also personified the enduring risks and resilience of the disease [2, 3]. Despite these setbacks, the possibility of a widespread EVD pandemic is now much farther in the distance as the outbreak transitions to its post-endemic phase. International efforts to finally put an end to this devastating chapter of Ebola instead focus on ongoing concerns of treating survivors and averting any potential further transmission, while exercising vigilance in surveillance, prevention, and maintaining response capacity.

Importantly, the EVD outbreak also marks a sentinel event in global public health, one that arguably requires a “shift” in how we approach governance for global health. Yet, the full repercussions of the outbreak on the future of historic international health institutions, such as the WHO, are only now starting to take shape. Hence, this piece provides a summary of the current international debate and discourse on global health governance reform measures post-EVD, focusing on the WHO and the future role of the United Nations in the broader global health architecture. This is accomplished by examining a series of reports issued by four high-level commissions/panels convened to specifically assess WHO’s performance and broader governance reform post-EVD. This includes recommendations from the WHO Interim Assessment panel established by the WHO Executive Board (Interim Panel); an external independent panel jointly convened by the Harvard Global Health Institute-London School of Hygiene & Tropical Medicine (“Harvard-LSHTM panel” comprised of members from academia, think tanks and civil society); the Commission on a Global Health Risk Framework for the Future (CGHRF) convened by the U.S. National Academy of Medicine (formerly the Institute of Medicine); and a separate High-Level Panel on the Global Response to Health Crises appointed by UN-Secretary General Ban Ki-moon (Kikwete Panel). It then discusses recent actions taken by WHO in response to calls for reform and also provides a critical assessment of how global health governance needs to “evolve” in order to modernize for the 21^st^ century.

## Discussion

### Is the “Evolution” of Global Health Governance Underway?

Influenza viruses evolve in two different ways including antigenic “drift” (e.g. small genetic mutations occurring continuously over time) and “shift” (e.g. major, abrupt changes/reassortment leading to different virus subtypes with high virulence and pandemic potential) [4]. This evolutionary process presents significant challenges in developing therapeutics and vaccines, as well as responding with appropriate public health measures, as viruses adapt to their environment and traverse multiple animal and human hosts. Similar to the “drift” that occurs in viral evolution, previous disease outbreaks of SARS (2002), H1N1/A (2009), MERS-CoV (2013), and the ever-looming threat of highly pathogenic influenza (e.g. H5N1), precipitated the current crisis in global health governance now occurring post-EVD [5–8]. Criticism has been swift, including strong statements from several heads of state, key civil society actors, national governments, and academia, opining on the need to pursue more radical reform measures, primarily focused on the future of WHO [9–13]. These developments could mark the beginnings of a “shift” in the evolution of global health governance.

#### Pre-Ebola reform

Calls to reform WHO are not new, but have grown incessantly louder in the wake of EVD [13, 14]. As the international public health agency charged with “the attainment by all people of the highest possible level of health”, WHO has faced many hurdles over the past two decades [15, 16]. Many of WHO’s challenges can be attributed to persistent budget limitations that have led to cuts in funding/staff and reallocation of resources from normative functions to discretionary programs highly influenced by donors [5, 6, 17]. Additional challenges arise from changing programmatic and Member State priorities, the formation of new global health initiatives (e.g. UNAIDS, GAVI, the Vaccine Alliance, PEPFAR, Stop TB Partnerships, and The Global Fund to Fight AIDS, Tuberculosis and Malaria) that often maintain parallel health systems and bypass traditional international governance structures, the rise of alternative channels/mechanisms of funding, and political inaction by Member States to pursue needed reforms identified as early as the 1990s [5, 6, 14–16, 18].

Previous international public health emergencies have also foreshadowed governance challenges that WHO would be forced to confront during the EVD outbreak. Specifically, the 2002 SARS outbreak, a novel coronavirus that spread to more than two dozen countries, marked a paradigm shift ushering in a new era of the globalized pathogen and demanded a modernization of WHO governance instruments and outbreak response processes [19, 20]. Though generally viewed as well managed due to an unprecedented international response coordinated by WHO and its Global Outbreak Alert and Response Network (GOARN), the SARS outbreak nevertheless exposed certain weaknesses [21]. Challenges included countries failing to report the threat of a potential outbreak with international implications, lack of sufficient “global” surveillance capacity, conflict between economic and trade considerations in public health emergencies, and global politics hindering WHO assistance [19, 21–24].

Most importantly, SARS also made it clear that the WHO’s International Health Regulations (IHR) were in need of an urgent update, leading to its revision in 2005, which required Member States to commit to minimum core public health systems, including surveillance, laboratory capacity, and emergency response capabilities [19, 25, 26]. The revision also granted WHO expanded authority by requiring Member States to proactively report potential international disease events and giving WHO the power to declare a “public health emergency of international concern” (PHEIC.) It also charged the agency with the difficult duty of balancing competing interests of trade, travel, human rights, and public health measures [19]. Fast forward to August 2014, when WHO issued its third-ever PHEIC for the EVD outbreak, and many of these challenges would “re-emerge” despite efforts to address them in the 2005 IHR revision.

Though a revision to the IHR was an important step post-SARS, more fundamental reform measures to address limitations associated with WHO’s governance and organizational structure have not been carried out as successfully. Proposals on how to pursue WHO reform post-SARS and pre-EVD have differed widely in scope and strategy. This includes reform proposals that have been structural, such as forming a new “Committee C” to engage a broader set of stakeholders (including civil society organizations); creating a “World Health Forum” for non-state actor engagement (a proposal rejected by Member States and criticized by civil society actors); splitting WHO into two separate technical and political entities; revising WHO’s constitution; and reforming WHO’s decentralized regional structure [6, 27–30]. Other reforms have focused on operational and financial aspects of WHO including: ensuring more sustainable operational financing by abolishing the zero-nominal growth requirement for member state contributions; allowing WHO to practice currency hedging, and establishing an ‘emergency fund’ [10, 31–33]. Still others have argued for additional powers/authority for WHO including: empowering WHO with additional normative "soft" and "hard" law instruments (e.g. 'Framework Convention on Global Health';) and complete "reinvention" of WHO's mandate, powers, and structures (see summary in Table 1) [10, 15, 34].

**Table 1:** Select WHO reform recommendations in the literature pre-EVD

Governance proposal	Description	Citation(s)
Committee C	Establishment of a new “Committee C” of WHA to debate major health initiatives and engage and coordinate across a broader array of global health stakeholders (including non-state actors.)	Silberschmidt G, Matheson D, Kickbusch I. Creating a committee C of the World Health Assembly. Lancet. 2008 May 3; 371(9623):1483–6. [27]
World Health Forum	Establishment of a new informal multistakeholder forum to engage non-state actors. This proposal was subsequently rejected by member states and also criticized by civil society actors	Commentary: Hawkes N. Re: “Irrelevant” WHO outpaced by younger rivals. BMJ 2015; 343(aug09 1):d5012–2. WHO website: http://www.who.int/dg/reform/en_who_reform_world_health_forum.pdf [28]
Splitting WHO	Dividing WHO secretariat functions into two different technical and political stewardship entities, with collaboration in areas that overlap.	Hoffman SJ, Rottingen J-A. Split WHO in two: strengthening political decision-making and securing independent scientific advice. Public Health 2014; 128(2):188–94. [6]
Revising WHO’s Constitution	Revising WHO’s constitution to fill the gaps in global governance as part of WHO reform process and for broader democratization of the agency.	Hoffman SJ, Rottingen J-A. Dark Sides of the Proposed Framework Convention on Global Health’s Many Virtues: A Systematic Review and Critical Analysis”. *Health & Human Rights Journal* 15(1): 117–134. [29]

Though the WHO reform process has been ongoing for decades crossing the tenure of several past WHO Director Generals, the formal reform process carried out by WHO immediately proceeding the EVD outbreak was limited in scope, primarily focused on incremental internal governance changes [14]. These included: reassessing future financing of WHO, setting the organization’s priorities in health, cutting its budget, drafting a framework for engagement with non-state actors, and implementation of other internal governance, programmatic, evaluation, accountability, and managerial reform measures [14, 35].

#### Post-Ebola reform

Post-EVD, WHO’s future is now at a critical juncture, as widespread criticism of WHO’s handling of the EVD outbreak has exposed fundamental weaknesses in the specialized agency’s ability to lead, coordinate, and mobilize an effective international response to the threat of a pandemic. With the stakes never higher, the urgency for WHO reform has been accelerated and is influenced by a collection of recommendations from four high-level panels/commissions that examined WHO’s performance during the EVD outbreak [13, 36, 37]. These include recommendations from the Interim Panel, the Harvard-LSHTM panel, the CGHRF, and the Kikwete Panel, which were reviewed and compared for: (1) proposals specifically addressing internal governance reforms or new mechanisms within WHO’s structure (not including reform proposals specific to the functioning of IHR, which deserve separate in-depth discussion); and (2) proposals focused on involvement and/or coordination from the United Nations on global health and health emergency activities (see summary of characteristics of Panels in Table 2).

**Table 2:** Characteristics of EVD High-level panels and commissions

Panel/commission name	Entity	Number of members/recommendations
WHO Interim Assessment Panel	Established by WHO Executive Board comprised of mix of independent experts	-Date issued: May 2015 −5 members −21 recommendations
Harvard-LSHTM Panel	Establishment by Harvard Global Health Institute and London School of Hygiene & Tropical Medicine primarily from academia, foundations, think tanks, and NGOs	-Date issued: November 2015 −22 members −10 recommendations
CGHR	Established as an independent commission with National Academy of Medicine as secretariat funded by foundations and agencies. Commission comprised of members from different countries, foundations, and entities.	-Date issued: January 2016 −17 members −26 recommendations
Kikwete Panel	Established by UN Secretary General comprised of political representatives of member states	-Date issued: January 2016 −6 members −27 recommendations
WHO Advisory Group on Reform of WHO’s Work in Outbreaks and Emergencies	Established by the WHO Director General to offer guidance on the organization’s emergency reform process. Group chaired by UN SG Special Envoy on Ebola and various members from UN agencies, NGOs, representatives of government health agencies, and others.	-Date issued: January 2016 −19 members −9 core recommendations in its second report

Note: Julio Frenk served on both the WHO Interim Panel and the CGHR. Lawrence Gostin and Gabriel Leung served on both the Harvard-LSHTM panel and CGHR

The first group to issue its recommendations was the WHO Interim panel comprised of independent experts appointed by WHO, who at the May 2015 68th World Health Assembly (WHA) delivered a report stating that the agency lacked the “capacity or organizational culture” to respond to emergency public health events [38]. The Interim Panel also concluded that WHO managed the crisis by prioritizing “good diplomacy” over necessary action, but offered no alternative to WHO, arguing instead that the agency should continue in its central role as the world’s lead health emergency response agency [38]. The panel recommended a set of reforms largely aimed at re-establishing WHO’s central role in health emergencies by advocating for: (a) strengthening of the IHR; (b) establishing a contingency fund for outbreak response; (c) formation of an independent Centre for Emergency Preparedness and Response (housed within WHO but overseen independently); (d) support for a WHO plan to develop a global health emergency workforce; and (e) WHO playing a more central role in R&D efforts for future health emergencies [38]. It also recommended the UN Secretary-General consider the appointment of a Special Representative or Special Envoy aimed at garnering greater political and financial support during a global health crisis, but did not recommend establishment of a permanent UN structure/mission. Importantly, many of these core proposals would set the framework for similar proposals expanded upon and carried forward by the other panels.

In November 2015, the Harvard-LSHTM panel published a set of 10 recommendations in the *Lancet*, which included several governance reform measures far more expansive than the first set of recommendations made by the Interim Panel. Reforms cover broader areas of global health governance and also include specific reform measures for WHO, all of which are grouped into four thematic areas of preventing, responding, conducting research, and governing the broader global system for disease outbreaks [39]. Reforms specific to WHO included: (a) creating a WHO dedicated independent centre for outbreak response; (b) formation of a politically insulated WHO Standing Emergency Committee for PHEIC declaration; (c) investing and strengthening global capacity to rapidly respond to outbreaks; (d) carrying out time-bound reforms to refocus and streamline WHO; (e) having WHO convene global stakeholders to develop a framework of norms and rules and a global financing facility for R&D relevant to disease outbreaks; and (f) instituting internal good governance reforms in exchange for more sustainable funding [39]. Broadly speaking the Harvard-LSHTM panel calls for more active engagement by WHO with the greater global community in managing infectious disease outbreaks, while also recommending that the agency scale back operational activities and instead focus on certain core functions [39]. Importantly, the panel also took the step of recommending the establishment of two structures by the UN Security Council: an Accountability Commission and a Global Health Committee that would independently assess outbreak response and elevate political attention to international health threats [39].

A few months later in January 2016, the CGHRF issued its own comprehensive report with an even more expansive list of 26 recommendations aimed at serving as a broader framework to address the “neglected” threat of infectious-disease crises. Recommendations were categorized under four domains of investment, building public health capabilities, strengthening surveillance, and accelerating R&D for pandemics [40]. Ten of these recommendations are specific to WHO, with some mirroring previous recommendations by the Interim and Harvard-LSHTM panels (e.g. formation of an independent Center funded by increased member state contributions, establishment of a contingency fund, and strengthening of the IHR.) [41]. The CGHRF generally advocated for strengthening WHO’s capacity to lead in pandemic preparedness and response by further reinforcing previous recommendation to create a WHO Center for Health Emergency Preparedness and Response, though independently overseen by a Technical Governing Board [41]. It also called for the involvement of the World Bank and International Monetary Fund (IMF) to help finance and strengthen implementation of IHR core capacities. Additionally, the CGHRF called for WHO to actively engage in other proposed governance structures that would oversee acceleration of R&D for pandemic preparedness and response (including the establishment of an independent Pandemic Product Development Committee) [41]. Finally, though the CGHRF report advocates for enhanced cooperation between WHO and regional, sub-regional, national governments, and non-state actors, it does not directly call for a UN leadership role, other than in the context of developing strategies for sustaining health systems capacity in fragile/failed states and during times of war.

Shortly thereafter, at the end of January 2016, the Kikwete Panel finalized its own report titled “Protecting Humanity from Future Health Crises”, recommending a final set of 27 measures to avert a future global pandemic, specifically noting that the risk of a highly pathogenic influenza virus was a chief concern [42]. Recommendations from the panel carry on similar themes to prior panel recommendations and are grouped into national, regional/sub-regional, and international-level recommendations, as well as sub-themes of development and health, R&D, financing, and follow-up and implementation recommendations. Chief among them included forming a Centre for Emergency Preparedness and Response within WHO, advancing full implementation of the IHR, securing appropriate financing for the WHO Centre and IHR compliance, and having WHO oversee the establishment of a fund and priority list to support R&D for neglected communicable diseases [42]. In addition, the panel strongly emphasized the need for a clear line of command within the UN system to coordinate a global response to a health and humanitarian emergency and more bodly  recommended the establishment of a High-level Council on Global Public Health Crises housed within the UN General Assembly [42]. Similar to the Interim Panel and CGHRF, the Kikwete Panel offered its strong endorsement of WHO as the “single” global health leader, but also noted that should the WHO fail to successfully reform or be empowered by its member states, that an “alternate” UN institutional response mechanism(s) might be necessary [42].

Collectively, these review panels, all governed by different stakeholders with varying operational mandates and perspectives, add increasing complexity to the urgent need for WHO reform post-EVD that the agency must now navigate (see Table 3 for a summary of reform recommendations summarized into themes of WHO reform and UN participation.) One proposal that had unanimous support was the formation of a WHO Centre for Emergency Preparedness and Response, which would be independently funded and governed but still housed within WHO [36, 37]. Other reform measures that garnered cross-panel support included the urgent need to increase WHO’s assessed contributions, developing mechanisms to enhance cooperation with non-state actors, strengthening global disease surveillance and IHR core capacities (including creating incentives/disincentives for IHR compliance,) and establishing better operational and policy coordination between WHO, UN agencies, and other global health partners [36, 37].

**Table 3:**
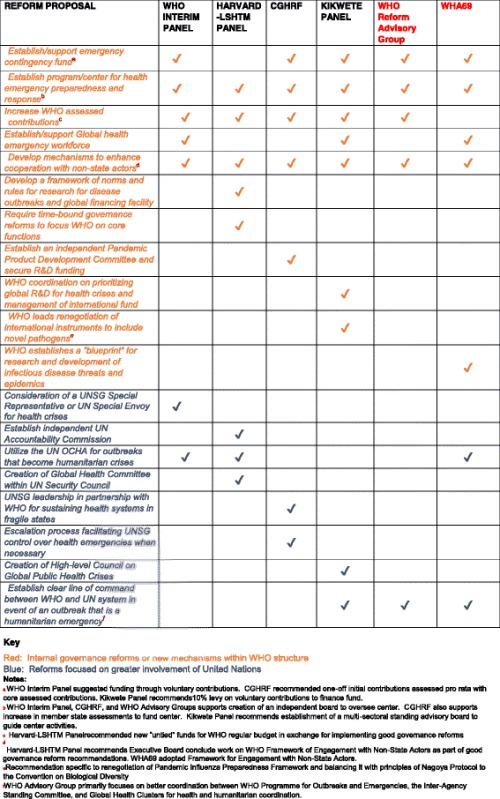
Matrix of WHO governance reform recommendations post-EVD

In response, to the myriad of recommendations set forth, the WHO, its Executive Board and its decision-making body, the WHA, were tasked with how to prioritize reforms, assess the feasibility and resources necessary to carry them out, and determining what reforms would be agreeable to all of its member states. Complicating this calculus is the fact that WHO’s current governance structure only allows formal participation by state actors, though the influence of powerful non-state actors (including those who provide the majority of funding to WHO through voluntary contributions) is unlikely to be completely silenced. The emergence of the Zika virus, which was declared a PHEIC event (subsequently removed November 2016), also has the potential to delay and/or significantly alter the pathway of post-EVD WHO reform. The emergence of Zika once again demonstrates that changes needed to ensure WHO can lead in averting the next health crisis are not currently in place [43]. Further, current WHO Director-General Dr. Margaret Chan’s tenure is coming to an end, meaning any long-term reforms will likely need to wait until her replacement is elected in 2017 [33, 37, 44].

## Conclusions

The 2014 EVD outbreak represents the re-emergence of an old and powerful infectious disease foe that has brought with it new urgency to address unresolved challenges in global health governance. At stake is the very nature and identity of WHO, which is feeling pressure to rediscover its role and relevance in a crowded and complex global health landscape populated by powerful nation states, large-scale bilateral health initiatives, multistakeholder partnerships, development banks, influential private foundations, and other UN agencies [14, 31, 45, 46]. How WHO will navigate the complexities of reform recommendations remains uncertain. What is clear is that the WHO needs to modernize in order to adapt to a new century of shared global health security, given ongoing threats from the emergence and re-emergence of infectious diseases, such as EVD and the Zika virus, and the inevitability of future outbreaks with pandemic potential.

### Prologue: reform responses by WHO

Despite the need for a revitalized WHO, reforms supported by WHO’s own high-level Advisory Group on Reform of WHO’s Work in Outbreaks and Emergencies (WHO Advisory Group, established by the WHO DG in July 2015), whose role was to provide guidance in relation to WHO’s emergency reform process (including the recommendations issued by the various panels), did not recommend some of the major changes advocated by the panels [47]. Instead, at the January 2016 WHO Executive Board meeting (coinciding with WHO declaring that EVD was no longer a PHEIC) , the WHO Advisory Group recommended critical enhancements focused on WHO internal outbreak and health emergency management, but did not recommend larger structural governance changes. Specifically, the group recommended the widely supported establishment of a centrally-managed, separate, dedicated WHO global Programme for Outbreaks and Emergencies headed by an Executive Director having its own budget and workforce that reports to the WHO DG [36, 47]. WHO would leverage the formation of this internal mechanism to also engage in other reform measures including ensuring greater health stakeholder collaboration, establishing better operational/ business processes during outbreaks, calling for an increase in member state assessed contributions for emergencies, capitalizing a contingency fund for emergency response, and improving resource mobilization, political engagement, accountability and external oversight [36, 47].

Following up on the WHO Advisory Group’s recommendations, at the 69th WHA in May 2016, Member States approved a plan formally establishing the WHO Health Emergencies Programme with a structure in many ways identical to that proposed by the WHO Advisory Group [48]. Accompanying its formation was an increase of $160 million to the existing WHO programme budget for WHO’s work in health emergencies, establishment by the DG of an independent oversight and advisory body, and the announcement of the appointment of Dr. Peter Salama (formerly with UNICEF) as the inaugural Executive Director [49]. Other reform recommendations that the WHO leadership has committed to carrying out or that are in the process of being implemented include the creation of a global health emergency workforce, strengthening implementation and monitoring of IHR core capacities, funding of the $100 million contingency fund for emergencies (via voluntary contributions including US$26.60 million received as of May 2016), and the development of an “R&D Blueprint” for accelerating R&D for health emergencies [49, 50].

Specific to the need for greater coordination and acceleration of R&D for vaccines, drugs, diagnostics, delivery systems, and other health technologies to avert a future epidemic when no existing medical countermeasures exists, the WHO published its “Plan of Action” for its R&D Blueprint on May 2016 [51]. This document presents the preliminary strategy for the WHO R&D Blueprint, as originally requested at the 68th WHA, and envisions a central convening and coordinating role for WHO in health R&D, a concept supported in different forms by all of the panels [51]. Though the Plan of Action envisions an inclusive and collaborative global approach to tackle the lack of R&D preparedness and access to treatment made evident during the EVD outbreak, it is unclear how this framework will be funded, implemented, and whether it will be aligned with existing governance structures and other related proposals. This includes existing structures such as the UN High-Panel on Access to Medicines and other financing and normative instruments currently being explored (including the WHO TDR Health Product Research & Development Fund and a proposed Global biomedical R&D treaty) [52].

More importantly, though the creation of WHO’s new Health Emergencies Programme represents a critical internal governance “drift”, structural reforms that would represent the needed governance “shift” to modernize WHO continue to lack the necessary political will and financial support for what would likely be a much more expensive reform process [14]. For example, restructuring of WHO’s current HQ/regional office/country office organizational structure has not been seriously considered, with the focus instead on bolstering staffing and support for WHO country offices and relying on the newly formed Health Emergencies Programme to enhance coordination [14, 38, 53]. The importance of restructuring WHO-led coordination (between WHO HQ, regional and country offices, and other support channels) was made evident by the lack of sufficient communication and coordination between WHO HQ and its largely autonomous regional office AFRO, a factor identified as contributing to the spread of EVD [13, 53].

Additionally, a needed increase and stabilization of WHO’s core budget (an 8% increase in voluntary contributions was approved at the 68th WHA but a 5% increase in member state assessments for the core budget was not; and a recent October 2016 WHO financing dialogue including a proposal by DG Chan to raise assessed contributions 10% received mixed reactions/support) has not been carried out, despite universal recommendation from all the panels on the need for more sustainable financing. Further, though the US$160 million increase in WHO’s budget to fund the Health Emergencies Progamme (which remains only 56% funded﻿ as of Oct 2016) is a needed investment, it falls far behind the CGHRF recommended annual investment of $4.5 billion to strengthen global infectious disease capacity []. Finally, though the WHO adopted a Framework for Engagement with Non-State Actors (FENSA) at the 69th WHA following 2 years of intergovernmental negotiations, the structure of the framework is limited to operational procedures and engagement management, and has also been criticized by certain stakeholders [14, 54, 55]. As such, it will likely fall short in establishing robust partnerships and creating a much needed space for formal interactions between WHO and critical non-state actors clearly needed during health emergencies.

### Greater Leadership by the UN in Global Health?

Growing recognition of the importance of global health and the unremitting threat of an emerging infectious disease outbreak may make it untenable to simply wait and hope for a sufficiently reformed WHO. Instead, the lasting legacy of the EVD outbreak may be its foreshadowing of a governance “shift” in formal sharing of the complex responsibilities of global public health and health security to other international structures, most notably various organs of the United Nations [36]. In September 2014, the UN authorized the first-ever UN emergency health mission, the UN Mission for Ebola Emergency Response (UNMEER), and thus became the central actor charged with mobilizing and coordinating resources across UN agencies, multiple states, and other partners working to stop EVD. UNMEER was established as a temporary measure to provide immediate financial, human resource, and logistic support for affected countries with the primary objectives of stopping the outbreak, treating the infected, ensuring access to essential services, creating stability, and preventing further escalation [56]. However, UNMEER’s participation has not been without criticism. In fact, reports by panels/commissions have noted that while UNMEER brought high-level political and financial support, coordination of the crisis became more difficult during its tenure in affected countries [38, 39]. Nevertheless, UNMEER’s creation brings a new dimension to global health governance as it is the first apparatus constructed to provide a singular UN system-wide approach in order to establish unity in combating a public health emergency. In addition to UNMEER, David Nabarro (﻿who acted as Chair of the WHO Advisory Group and who is also a current candidate for the new WHO DG) was appointed as the UN Secretary-General's Special Envoy on Ebola in August 2014, for the purpose of providing strategic and policy direction to improve the international response to EVD.

UNMEER’s creation, though not viewed as entirely successful, may serve as a precursor for what future global health governance structures could look like in the absence of an adequately strengthened and empowered WHO. Hints of this potential governance “shift” manifested in different panel recommendations, with all panels calling for some form of increased involvement by the larger UN system. Specifically, the Interim Panel called for the appointment of a Special Representative of the Secretary-General or a UN Special Envoy for high-level global health threats, but stopped short of recommending the establishment of a full UN mission [57]. The Harvard-LSHTM report concluded that an independent UN Accountability Commission was needed to assess worldwide responses to outbreaks and also recommended the creation of a Global Health Committee as part of the UN Security Council [39]. The CGHRF called for better communication and collaboration mechanisms between WHO and the UN (including an escalation process to transfer control of emergencies from WHO to the UN Secretary-General,) and also specified that the UN Secretary-General should lead in developing strategies for sustaining health system capacities in fragile/failed states, though it did not support the creation of a new UN entity [41]. The UN’s own Kikwete Panel specifically recommended the creation of a High-level Council on Global Public Health Crises housed within the General Assembly made up of 45–50 political representatives of member states in order to ensure political accountability and reform implementation [42]. Germany's Chancellor Angela Merkel’s keynote speech at 68th WHA was even more direct, stating that WHO cannot tackle Ebola or global health on its own, and that its cooperation with the broader UN system and World Bank is a critical component of any reform moving forward [58, 59].

### A UN High-Panel on Global Health: 21^st^ Century Global Health Governance?

**Fig. 1:**
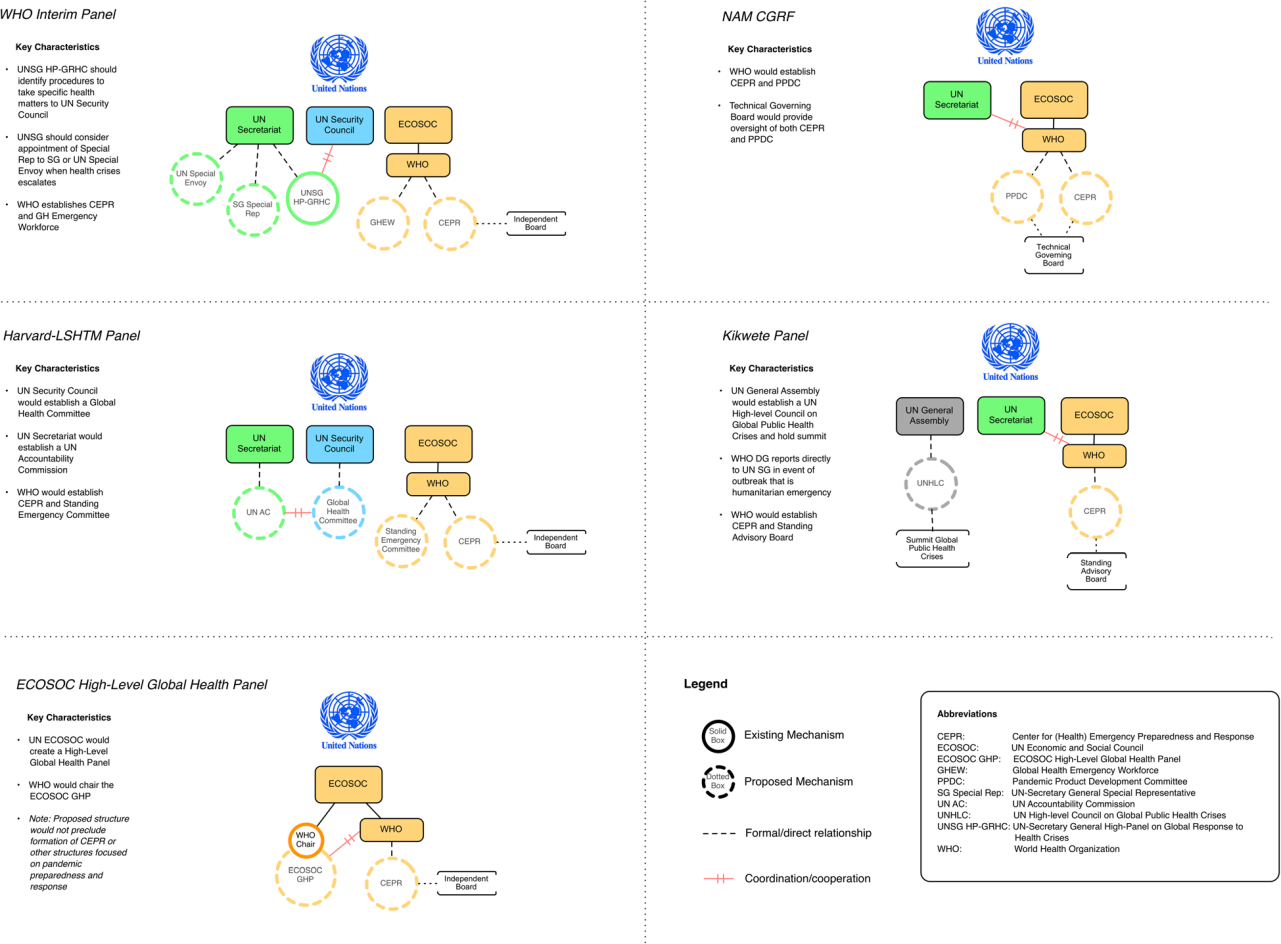
Visualization of Global Health Governance Reform Structures. Attached as separate file

Though calls for greater involvement by the UN in global health have accelerated following the EVD outbreak, recommendations for a permanent UN global health structure are not new. As early as 2012, this author specifically advocated for the formation of a UN Global Health Panel chaired by WHO, which could alternatively be housed within the UN Economic and Social Council (ECOSOC) - a central mechanism of the UN system that has been active in global health issues (including developing the UN Sustainable Development Goals) and is the principal UN organ coordinating work across its 14 specialized agencies [45]. ECOSOC represents an optimal space to establish cooperation, coordination, and policy coherence across the entire UN system, its technical agencies, and its respective stakeholder networks on key global health challenges, including health emergencies (see Fig. 1 for visualization of governance recommendations by the panels and the UN Global Health Panel recommendation) [45].

An ECOSOC UN Global Health Panel would meet many of the needs identified in post-EVD governance reform discussions, including acting as a permanent central body to coordinate global health efforts across the entire UN system (including alignment with the SDGs), allowing engagement with a broader array of stakeholders and creating a "safe space" for these interactions through its partnership initiatives and forums, and could be designed in a way that would afford it greater political visibility as well as increased accountability by having it report directly to the UN General Assembly [37, 45, 60–62]. Similarly, the creation of a Global Health Committee contained within the UN Security Council - as recommended by the Harvard-LSHTM panel - could also elevate health emergencies to the highest levels of political attention and raise awareness among key decisionmakers. However, the highly political nature of the Security Council could also result in public health priorities being stymied by larger and more influential political, security, and core foreign policy dynamics, as has been the case in other health diplomacy outcomes [63–65]. Further, the High-level Council proposed by the Kikwete Panel would not implicitly include important multistakeholder representation from international public health agencies, non-state actors, NGOs, civil society, and other health actors, instead relying upon the exclusive participation of member state political representatives.

More fundamentally, critical in assessing whether any UN global health apparatus will work as a central pillar in the future architecture of global health governance is defining the appropriate roles and responsibilities of WHO with respect to other UN institutions that are already actively participating in global health [60]. This is necessary in order to avoid duplication and ensure effective coordination, a challenge highlighted by the temporary and emergency nature of the UNMEER governance intervention during EVD. This would require focusing a proposed UN global health panel’s structure on galvanizing high-level political and financial attention to a health emergency/outbreak, while also creating a permanent policy and governance environment conducive to multistakeholder partnership building, coordination, and mobilization of resources that is beyond the scope of WHO’s current operational mandates and capacity.

However, this structure would also need to leverage the unique strengths of WHO, specifically allowing the embattled agency to focus on its indispensable role as the world’s chief technical health agency by imbuing it as the Chair of the proposed structure and having the newly established WHO Health Emergencies Programme take a leading role when addressing issues related to health emergencies and outbreask with panademic potential [45]. Though some may view a UN-based structure as usurping WHO’s credibility and leading to duplication, by positioning WHO as the chair of a UN Global Health Panel, such a structure could serve the dual role of reestablishing WHO’s relevance and strengthening its response capabilities [66–68]. This would be accomplished by enabling WHO with high-level political representation and access to all the resources at the UN system’s disposal.

Through this structure, WHO could then establish clear and delineated roles for other actors when preparing for and responding to health emergencies. It could then act as both the primary coordination mechanism and provide technical support to other UN institutions, such as the World Bank, UN Development Programme, UNICEF, UNAIDS, UN Environmental Programme, UN Population Fund, UN Office of Drugs and Crime, Food and Agriculture Organization, many of whom were active participants in the fight against EVD [45]. The design of the structure should also leverage existing inter-agency structures already established between WHO, the UN and other health partners (including the Global Health Cluster and Inter-Agency Standing Committee that both focus on humanitarian and health issues.) [13]. In this sense, a UN global health panel apparatus could act as a hybrid multistakeholder global health body bringing together the respective organizational and partnership networks available between the UN system and WHO. This would enable more robust engagement with key civil society actors, NGOs, foundations, civil-military cooperation, and private sector actors that will be critical in the prevention, detection, and response to future health emergencies and potential pandemics.

Finally, by placing global health, health emergencies, and infectious disease governance at the top of the UN hierarchy, the opportunity to establish greater policy coherence across the entire landscape of global governance mandates and instruments could be better realized by a UN Global Health Panel. This includes ensuring that all member states, UN agencies, and non-state partners are aligned under SDG goals 3.b (supporting R&D for infectious diseases), 3.c (ensuring a sustainable health workforce in developing countries) and 3.d (strengthening capacity for risk reduction and management of global health risks), all factors critical in preventing and responding to infectious disease outbreaks. This in turn could translate to better funding, strengthening, and implementation of the IHR and the WHO R&D Blueprint, while also supporting wider adoption of the WHO Global Code of Practice on the International Recruitment of Health Personnel [69, 70]. Additionally, other global health governance mechanisms can also be championed at highest levels of political engagement, including proposed instruments such as the biomedical R&D treaty and the Framework Convention for Global Health, which could bolster the normative powers of WHO [29, 71, 72]. This could establish a strengthened global health governance system, a step that is needed to ensure that global health and infectious disease outbreaks are given their rightful priority in the ec﻿helons of foreign policy and international affairs.

## Summary

Only time will tell if the legacy of EVD will include a WHO that has the full support of the international community and is capable of leading human society in this brave new era of globalization and health. Despite current uncertainty, the time is ripe for a radical “shift” in global health governance by recognizing that complex global health challenges can no longer be borne by WHO alone. Instead, global health demands a UN systems wide approach lead by a permanent UN Global Health apparatus combined with WHO reforms that are fair to the agency’s current funding and capacity limitations while also leveraging its core strengths as the world’s preeminent international health body. The urgency for these reforms comes at a time when global health is increasingly becoming a foci for the security, economic, social, political and health interests of all nation states, necessitating greater shared leadership. In this sense, the impact of the EVD outbreak is likely to go far beyond the immense human suffering and lives lost during this tragedy, extending to the very foundation of how we approach governance for global health in the 21^st^ century.

## Abbreviations

AFRO: World Health Organization Regional Office for Africa
CGHRF: Commission on a Global Health Risk Framework for the Future
DG: Director General
ECOSOC: United Nations Economic and Social Council
EVD: Ebola virus disease outbreak
FAO: Food and Agriculture Organization of the United Nations
FENSA: WHO Framework for Engagement with Non-State Actors
GOARN: WHO Global Outbreak Alert and Response Network
Harvard-LSHTM panel: External independent panel jointly convened by the Harvard Global Health Institute-London School of Hygiene & Tropical Medicine
Interim Panel: WHO Interim Assessment panel established by the WHO Executive Board
IHR: International Health Regulations
IMF: International Monetary Fund
Kikwete Panel: High-Level Panel on Global Response to Health Crises appointed by UN-Secretary General Ban Ki-moon
PHEIC: Public Health Emergency of International Concern
UN: United Nations
UNAIDS: Joint United Nations Programme on HIV/AIDS
UNDP: United Nations Development Programme
UNEP: United Nations Environment Programme
UNFPA: United Nations Population Fund
UNICEF: United Nations Children’s Emergency Fund
UNMEER: United Nations Mission for Ebola Emergency Response
UNODC: United Nations Office on Drugs and Crime
WHA: World Health Assembly
WHO: World Health Organization
WHO Advisory Group: High-level Advisory Group on Reform of WHO’s Work in Outbreaks and Emergencies
WHO HQ: World Health Organization Head Quarters (Geneva, Switzerland)

## References

[1] WHO. Statement on the 9th meeting of the IHR Emergency Committee regarding the Ebola outbreak in West Africa [Internet]. WHO. World Health Organization; 2016 [cited 2016 Apr 13]. Available from: http://www.who.int/mediacentre/news/statements/2016/end-of-ebola-pheic/en/. Accessed 20 Nov 2016.

[2] WHO. Ebola Situation Report - 2 March 2016 [Internet]. apps.who.int. 2016 [cited 2016 Mar 10]. Available from: http://apps.who.int/ebola/current-situation/ebola-situation-report-2-march-2016.

[3] Mackey TK (2016). Lessons from LIberia: Global Health Governance in the Post-Ebola Paradigm. Global Health Governance.

[4] Treanor J (2004). Influenza Vaccine — Outmaneuvering Antigenic Shift and Drift. N Engl J Med.

[5] Sridhar D, Frenk J, Gostin L, Moon S (2014). Global rules for global health: why we need an independent, impartial WHO. BMJ.

[6] Hoffman SJ, Rottingen J-A (2014). Split WHO in two: strengthening political decision-making and securing independent scientific advice. Public Health.

[7] KICKBUSCH I (2015). Reddy KS.

[8] Statement from Assistant Secretary Jimmy Kolker at the Special Session of the WHO Executive Board on Ebola, Sunday, January 25, 2015 [Internet]. globalhealth.gov. 2015 [cited 2015 Jul 22]. Available from: https://geneva.usmission.gov/2015/01/30/remarks-by-assistant-secretary-kolker-at-who-special-session-on-ebola/. Accessed 21 Nov 2016.

[9] Siedner MJ, Gostin LO, Cranmer HH, Kraemer JD. Strengthening the detection of and early response to public health emergencies: lessons from the West African Ebola epidemic. PLoS Med. Public Library of Science; 2015;12:e1001804. 10.1371/journal.pmed.1001804 PMC4371887 25803303

[10] Gostin LO (2015). Reforming the World Health Organization after Ebola. JAMA.

[11] Garrett L (2015). Ebola’s Lessons. Foreign Aff.

[12] Kupferschmidt K (2015). Global health. Report prescribes strong medicine for WHO. Science.

[13] Checchi F, Waldman RJ, Roberts LF, Ager A, Asgary R, Benner MT, et al. World Health Organization and emergency health: if not now, when? BMJ : British Medical Journal. British Medical Journal Publishing Group; 2016;352:i469. 10.1136/bmj.i469 26821569

[14] Yach D (2016). World Health Organization Reform—A Normative or an Operational Organization?. Am J Public Health.

[15] Lee K, Pang T (2014). WHO: retirement or reinvention?. Public Health.

[16] Lidén J (2014). The World Health Organization and Global Health Governance: post-1990. Public Health.

[17] Collier R (2012). World Health Organization reform languishes. Can Med Assoc J.

[18] Wibulpolprasert S, Chowdhury M (2016). World Health Organization: Overhaul or Dismantle?. Am J Public Health.

[19] Mackey TK, Liang BA (2012). Lessons from SARS and H1N1/A: employing a WHO-WTO forum to promote optimal economic-public health pandemic response. J Public Health Policy.

[20] Baker MG, Fidler DP (2006). Global public health surveillance under new international health regulations. Emerg Infect Dis.

[21] Institute of Medicine (US) Forum on Microbial Threats, Knobler S, Mahmoud A, Lemon S, Mack A, Sivitz L, et al. Learning from SARS: Preparing for the Next Disease Outbreak: Workshop Summary. Washington (DC): National Academies Press (US); 2004. 22553895

[22] Chan L-H, Chen L, Xu J. China's Engagement with Global Health Diplomacy: Was SARS a Watershed? PLoS Med. Public Library of Science; 2010;7:e1000266. 10.1371/journal.pmed.1000266 PMC2860492 20436959

[23] Liang BA, Mackey TK. Preparing for health diplomacy negotiations – global governance and the case of Taiwan, WHO, and SARS. Rosskam E, Kickbusch I, editors. Negotiating and Navigating Global Health: Case Studies in Global Health Diplomacy. London: Scientific Press.

[24] Herington J, Lee K (2014). The limits of global health diplomacy: Taiwan's observer status at the world health assembly. Global Health..

[25] Gates B. The Next Epidemic — Lessons from Ebola. N Engl J Med. 2015; 372:1381-84. 10.1056/NEJMp1502918 25853741

[26] Heymann DL, Chen L, Takemi K, Fidler DP, Tappero JW, Thomas MJ (2015). Global health security: the wider lessons from the west African Ebola virus disease epidemic. Lancet.

[27] Silberschmidt G, Matheson D, Kickbusch I (2008). Creating a committee C of the World Health Assembly. Lancet.

[28] Hawkes N (2015). “Irrelevant” WHO outpaced by younger rivals. BMJ.

[29] Hoffman SJ, Rottingen J-A (2013). Dark sides of the proposed Framework Convention on Global Health’s many virtues: A systematic review and critical analysis. Health Hum Rights.

[30] Gostin LO (2015). World health organization reform: lessons learned from the Ebola epidemic. Hastings Cent Rep.

[31] Pang T, Garrett L (2012). The WHO must reform for its own health. Nat Med.

[32] Mackey TK, Novotny TE. Improving United Nations Funding to Strengthen Global Health Governance: Amending the Helms–Biden Agreement. Global Health Governance. 2012.

[33] Frenk J. Finance and Governance: Critical Challenges for the Next WHO Director-General. http://dx.doi.org/10.2105/AJPH.2016.303399. American Public Health Association; 2016. pp. 1906–7. 10.2105/AJPH.2016.303399 PMC5055779 27715290

[34] Ooms G, Marten R, Waris A, Hammonds R, Mulumba M, Friedman EA (2014). Great expectations for the World Health Organization: a Framework Convention on Global Health to achieve universal health coverage. Public Health.

[35] WHO. WHO reform process [Internet]. who.intaboutwhoreformprocessen. World Health Organization; 2016 [cited 2016 Mar 10]. Available from: http://www.who.int/about/who_reform/process/en/.

[36] Gostin LO, Tomori O, Wibulpolprasert S, Jha AK, Frenk J, Moon S, et al. Toward a Common Secure Future: Four Global Commissions in the Wake of Ebola. PLoS Med. Public Library of Science; 2016;13:e1002042. 10.1371/journal.pmed.1002042 PMC4873000 27195954

[37] Sridhar D, Kickbusch I, Moon S, Dzau V, Heymann D, Jha AK, et al. Facing forward after Ebola: questions for the next director general of the World Health Organization. BMJ : British Medical Journal. British Medical Journal Publishing Group; 2016;353:i2666. 10.1136/bmj.i2666 27193334

[38] Report of the Ebola Interim Assessment Panel [Internet]. WHO. 2015 [cited 2015 Jul 25]. Available from: http://www.who.int/csr/resources/publications/ebola/report-by-panel.pdf?ua=1.

[39] Moon S, Sridhar D, Pate MA, Jha AK, Clinton C, Delaunay S, et al. Will Ebola change the game? Ten essential reforms before the next pandemic. The report of the Harvard-LSHTM Independent Panel on the Global Response to Ebola. The Lanceancet. 2015;386(10009):2204–21. 10.1016/S0140-6736(15)00946-0 PMC7137174 26615326

[40] Sands P, Mundaca-Shah C, Dzau VJ (2016). The Neglected Dimension of Global Security — A Framework for Countering Infectious-Disease Crises. N Engl J Med.

[41] National Academy of Medicine (2016). The Neglected Dimension of Global Security: A Framework to Counter Infectious Disease Crises.

[42] Protecting Humanity from Future Health Crises: Report of the High-level Panel on the Global Response to Health Crises [Internet]. United Nations. 2016 [cited 2016 Apr 13]. Available from: http://www.un.org/News/dh/infocus/HLP/2016-02-05_Final_Report_Global_Response_to_Health_Crises.pdf. Accessed 20 Nov 2016.

[43] Lucey DR, Gostin LO (2016). The Emerging Zika Pandemic: Enhancing Preparedness. JAMA.

[44] Davies SC, Akksilp S, Takemi K, Matsoso P, da Silva JB (2016). The future leadership of WHO. Lancet.

[45] Mackey TK, Liang BA (2013). A United Nations Global Health Panel for Global Health Governance. Soc Sci Med.

[46] Pablos-Mendez A, Baker S. A New Leader for a New World Health. Am J Public Health. 2016; 106(11): 1907-8. http://dx.doi.org/10.2105/AJPH.2016.303474. 10.2105/AJPH.2016.303474 PMC5055804 27715291

[47] ADVISORY GROUP ON REFORM OF WHO’S WORK IN OUTBREAKS AND EMERGENCIES SECOND REPORT [Internet]. WHO. 2016 [cited 2016 Jun 6]. Available from: http://www.who.int/about/who_reform/emergency-capacities/advisory-group/second-report.pdf?ua=1. Accessed 20 Nov 2016.

[48] World Health Assembly agrees new Health Emergencies Programme [Internet]. WHO. World Health Organization; 2016 [cited 2016 Jun 6]. Available from: http://www.who.int/mediacentre/news/releases/2016/wha69-25-may-2016/en/.

[49] Progress Report on the Development of the WHO Health Emergencies Programme [Internet]. WHO. 2016 [cited 2016 Jun 6]. Available from: http://www.who.int/about/who_reform/emergency-capacities/who-health-emergencies-programme-progress-report-march-2016.pdf?ua=1. Accessed 20 Nov 2016.

[50] Kieny MP, Rottingen JA, Farrar J. The need for global R&D coordination for infectious diseases with epidemic potential. Lancet. 2016;288(10043): 460-61. 10.1016/S0140-6736(16)31152-7 PMC7133575 27507751

[51] WHO. An R&D Blueprint for Action to Prevent Epidemics: Plan of Action [Internet]. WHO. 2016 [cited 2016 Oct 21]. Available from: http://www.who.int/csr/research-and-development/WHO-R_D-Final10.pdf. Accessed 20 Nov 2016.

[52] UN. Report of the United Nations Secretary General's High-Level Panel on Access to Medicines [Internet]. unsgaccessmeds.org. 2016 [cited 2016 Oct 21]. Available from: http://www.unsgaccessmeds.org/final-report/. Accessed 20 Nov 2016.

[53] Kamradt-Scott A (2016). WHO’s to blame? The World Health Organization and the 2014 Ebola outbreak in West Africa. Third World Quarterly. Routledge.

[54] WHO. Framework of engagement with non-State actors: draft resolution [Internet]. apps.who.int. 2016 [cited 2016 Jun 6]. Available from: http://apps.who.int/gb/ebwha/pdf_files/WHA69/A69_ACONF11-en.pdf. Accessed 20 Nov 2016.

[55] Saez C. WHA Gets First UN Framework Managing Non-State Actors; Countries Satisfied, Actors Concerned [Internet]. 2016 [cited 2016 Jun 6]. Available from: https://saudeglobal.org/2016/06/03/wha-gets-first-un-framework-managing-non-state-actors-countries-satisfied-actors-concerned-by-catherine-saez/. Accessed 20 Nov 2016.

[56] UN Mission for Ebola Emergency Response (UNMEER) [Internet]. ebolaresponse.un.org. 2014 [cited 2016 Apr 14]. Available from: http://ebolaresponse.un.org/un-mission-ebola-emergency-response-unmeer. Accessed 20 Nov 2016.

[57] WHO response to the Ebola Interim Assessment Panel report [Internet]. WHO. World Health Organization; 2015 [cited 2015 Jul 25]. Available from: http://www.who.int/mediacentre/news/statements/2015/ebola-panel-report/en/. Accessed 20 Nov 2016.

[58] Garrett L. The Ebola Review, Part II [Internet]. foreignpolicy.com. 2015 [cited 2015 Jul 22]. Available from: https://foreignpolicy.com/2015/06/06/ebola-review-part-ii-g-7-merkel-world-health-organization/. Accessed 20 Nov 2016.

[59] Horton R (2015). Offline: An irreversible change in global health governance. Lancet.

[60] Mackey TK, Liang BA. Response to comments on “A United Nations Global Health Panel for Global Health Governance”. Soc Sci Med. 2012;76:24–7. 10.1016/j.socscimed.2012.09.038 23121855

[61] Dussault G (2016). The Election of the Next World Health Organization Director-General Explained to a Visitor From Mars. Am J Public Health.

[62] Horton R, Samarasekera U (2016). WHO's Director-General candidates: visions and priorities. The Lancet.

[63] Feldbaum H, Michaud J (2010). Health diplomacy and the enduring relevance of foreign policy interests. PLoS Med.

[64] Setayesh S, Mackey TK (2016). Addressing the impact of economic sanctions on Iranian drug shortages in the joint comprehensive plan of action: promoting access to medicines and health diplomacy. Global Health.

[65] Mackey TK, Strathdee SA (2015). Responding to the public health consequences of the Ukraine crisis: an opportunity for global health diplomacy. J Int AIDS Soc.

[66] Sridhar D (2013). Coordinating the UN System: lessons from UNAIDS: a commentary on Mackey. Soc Sci Med.

[67] Dussault G (2013). A United Nations Global Health Panel for Global Health Governance: a commentary on Mackey. Soc Sci Med.

[68] Hein W (2013). A United Nations Global Health Panel for Global Health Governance: a commentary on Mackey. Soc Sci Med.

[69] Taylor AL, Hwenda L, Larsen B-I, Daulaire N (2011). Stemming the brain drain--a WHO global code of practice on international recruitment of health personnel. N Engl J Med.

[70] Fischer JE, Katz R (2013). Moving forward to 2014: global IHR (2005) implementation. Biosecur Bioterror.

[71] Gostin LO (2012). A Framework Convention on Global HealthHealth for All, Justice for AllA Framework Convention on Global Health. JAMA.

[72] Balasegaram M, Bréchot C, Farrar J, Heymann D, Ganguly N, Khor M (2015). A Global Biomedical R&D Fund and Mechanism for Innovations of Public Health Importance. PLoS Med.

